# Low power implementation of high frequency SiPM readout for Cherenkov and scintillation detectors in TOF-PET

**DOI:** 10.1088/1361-6560/ac8963

**Published:** 2022-09-26

**Authors:** Joshua W Cates, Woon-Seng Choong

**Affiliations:** Lawrence Berkeley National Laboratory, Berkeley, CA, United States of America

**Keywords:** time-of-flight PET, coincidence time resolution, silicon photomultipliers, high frequency readout, low power readout, Cherenkov detectors, scintillation detectors

## Abstract

State-of-the-art (SoA) electronic readout for silicon photomultiplier (SiPM)-based scintillation detectors that demonstrate experimental limits in achievable coincidence time resolution (CTR) leverage low noise, high frequency signal processing to facilitate a single photon time response that is near the limit of the SiPMs architecture. This readout strategy can optimally exploit fast luminescence and prompt photon populations, and promising measurements show detector concepts employing this readout can greatly advance PET detector CTR, relative to SoA in clinical systems. However, the technique employs power hungry components which make the electronics chain impractical for channel-dense time-of-flight (TOF)-PET detectors. We have developed and tested a low noise and high frequency readout circuit which is performant at low power and consists of discrete elements with small footprints, making it feasible for integration into TOF-PET detector prototypes. A 3 × 3 mm^2^ Broadcom SiPM with this readout chain exhibited sub-100 ps single photon time resolution at 10 mW of power consumption, with a relatively minor performance degradation to 120 ± 2 ps FWHM at 5 mW. CTR measurements with 3 × 3 × 20 mm^3^ LYSO and fast LGSO scintillators demonstrated 127 ± 3 ps and 113 ± 2 ps FWHM at optimal power operation and 133 ± 2 ps and 121 ± 3 ps CTR at 5 mW. BGO crystals 3 × 3 × 20 mm^3^ in size show 271 ± 5 ps FWHM CTR (1174 ± 14 ps full-width-at-tenth-maximum (FWTM)) at optimal power dissipation and 289 ± 8 ps (1296 ± 33 ps FWTM) at 5 mW. The compact and low power readout topology that achieves this performance thereby offers a platform to greatly advance PET system CTR and also opportunities to provide high performance TOF-PET at reduced material cost.

## 1. Introduction

Precise estimation of 511 keV photon arrival time at the detector elements of positron emission tomography systems allows their origin to be accurately estimated within a region along system response lines dictated by the coincidence time resolution (CTR) of the detectors. Incorporating this information into the image reconstruction enables time-of-flight (TOF)-PET, which can produce significant gains in reconstructed image signal-to-noise-ratio (SNR) by localizing annihilation along lines of response (Conti 2008). The magnitude of this SNR boost scales with improved CTR, and state of the art (SoA) clinical systems achieve approximately 200–400 picoseconds (ps) FWHM CTR, depending upon system design (Miller et al 2015, Hsu et al 2017, van Sluis et al 2019). This CTR performance provides 3–6 cm of event localization and an estimated 3.7–2.6 fold improvement in reconstructed image SNR (as calculated by estimated SNR gain from TOF technique in Conti (2008)), relative to reconstruction with no TOF information incorporated. Improving time resolution in TOF-PET continues to be a subject of intense study as several research efforts strive to improve system-level CTR far below the 100 ps threshold, towards the limit of event localization dictated by positron range in tissue (Lecoq et al 2020), at approximately 10 ps for ^18^F.

Low noise and high frequency front-end electronic readout of silicon photomultipliers (SiPMs) (Cates et al 2018, Gundacker et al 2019) is a primary method for demonstrating the latest experimental limits in achievable CTR with fast scintillation detectors and detection media that derive 511 keV photon interaction time estimation from small populations of promptly emitted optical photon signatures (Gundacker et al 2020,^2021^, Kratochwil et al 2021, Loignon-Houle et al 2021). By minimizing the influence of electronic noise jitter on signal processing, this readout topology can provide single photon time resolution (SPTR) for large area SiPMs that is near the limit dictated by the device architecture (Cates et al 2018), and it also provides excellent SNR for leading edge time estimators. The downside to this approach has been the high power consumption required from the developed readout scheme (⩾200 mW for a single channel of readout, in some implementations). In addition, the footprint of this readout circuit can be relatively large. Thus, without optimization of power consumption and electronic footprint, the advances in achievable CTR demonstrated with this readout topology cannot be practically realized in multichannel TOF-PET detector readout.

To address this, we have assembled and tested a compact, low power circuit to create electronic readout topologies that are tractable for integration into large area PET detector modules, aiming to realize the excellent benchtop performance capabilities demonstrated with high frequency chains in prototype PET imaging systems. Newer generations of low noise amplifiers leveraging SiGe bipolar transistors (such as SiGe:C (Knoll et al 2000)), more commonly used in telecommunications applications, can offer ultra-wide bandwidth (10 s of GHz) and high gain (10–30 dB per element) at very low lower noise metrics (~1 dB). Since the frequency domain relevant to radiation detection instrumentation is much narrower than these newer devices achieve with optimal power supply, operating voltage can be significantly reduced and still maintain adequate frequency response, along with fast rising edge slew and reduced noise, without affecting operability or stability. In this way, these low noise amplifiers can be implemented in a readout that provides the benefits of high frequency SiPM readout at greatly reduced power consumption.

In this work, we designed and implemented micro-balun transformers and low power, high performance, SiGe:C monolithic microwave integrated circuit (MMIC) amplifiers into a test circuit which includes a commercial, near-ultra-violet (near-UV) sensitive SiPM. We evaluated achievable SPTR with the SiPM and readout combination as a function of power consumption. CTR for fast scintillators and crystals with moderate Cherenkov yield was also studied with the readout as a function of the readout circuit’s power consumption. We show the achievable performance with the test circuit is essentially identical to that achieved with previous implementations of high frequency SiPM readout that require much higher power supply for operation.

## 2. Materials and experimental methods

Two major hurdles to translating the excellent CTR that low noise and high frequency front-end signal processing has demonstrated for various Cherenkov and scintillation detectors are the footprint and high power consumption of balun transformer and RF amplifiers, respectively. Concerning the footprint of the balun transformer, micro-balun transformers used in transmission line impedance matching, such as those listed in table 1, can be used to reduce that component’s impact on readout footprint. These components integrate wound cores into small packages which are slightly larger than 1 millimeter (mm) on each side and can be submm in height. Their frequency ranges and port impedances are also generally well matched to fast front-end SiPM readout. Current generation SiGe and SiGe:C MMICs, such as the examples listed in table 2, also bring wide frequency response, high gain, and fast response at relatively low current supply. Moreover, they are typically integrated into small packages for flexible integration into compact telecommunications devices.

### 2.1. Electronic readout circuit

**Figure 1.**
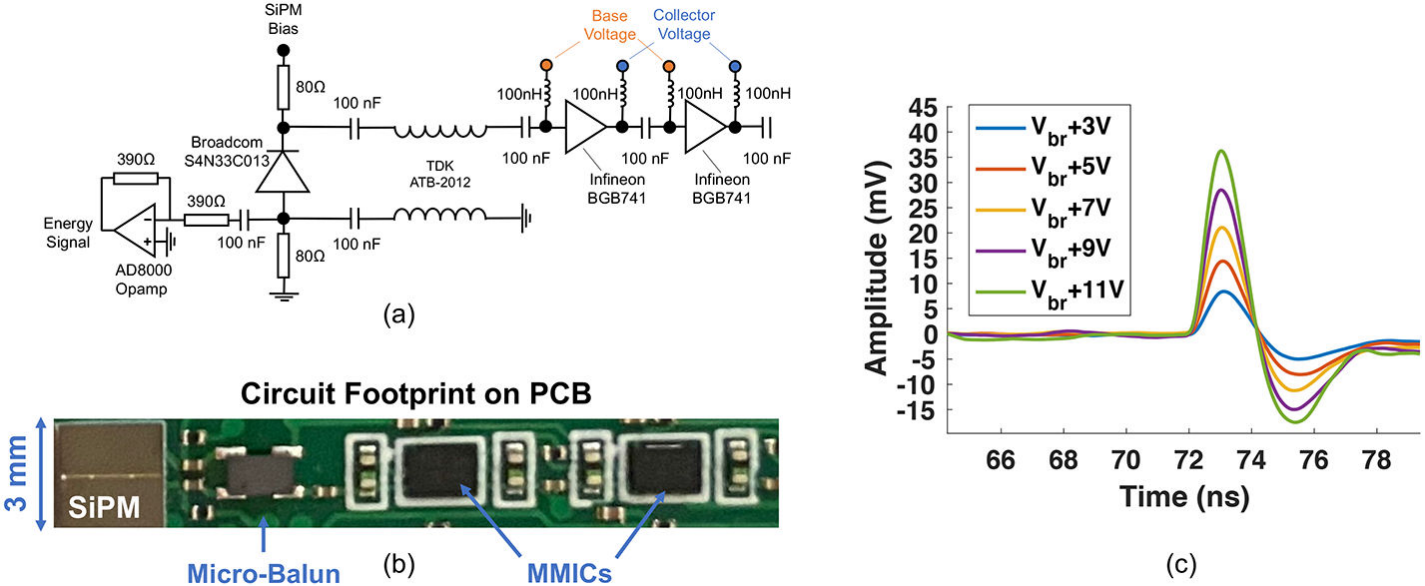
A schematic of the low power, low noise and high frequency test circuit is shown in (a), with layout on PCB shown in (b). Example single photon pulses from the test circuit with laser illumination is shown as a function of overvoltage in (c). Breakdown voltage for these SiPMs is specified as 26.9 ± 0.18 V. Thus, the overvoltage range shown in (c) corresponds to a voltage range of 30–38 V applied to the SiPM.

A high frequency, low noise SiPM readout circuit was implemented onto a printed circuit board with electronic schematic outlined in figure 1(a), comprising a 3 × 3 mm^2^ Broadcom AFBR-S4N33C013 SiPM coupled differentially to a TDK ATB2012 micro-balun transformer and a cascade of two Infineon BGB741L7ESD low noise, MMIC amplifiers. As shown in tables 1 and 2, both of these components have good frequency response and impedance matching (for the micro-balun transformer), achievable gain, noise metric, required power supply, and small footprint. The BGB741L7ESD also has current adjustment and biasing terminals that were attractive for circuit layout and evaluation. The selected micro-balun transformer and MMIC are also widely available commercially and suggested for new designs by their respective manufacturers. Although we did not experimentally compare multiple combinations of the components listed in these tables, we note that all of the components listed in tables 1 and 2 are good choices for a low noise, high frequency SiPM readout implementation. We provide a list of appropriate components in tables 1 and 2 to highlight the fact that there are a number of commercial devices that could be implemented to adapt such a circuit into a prototype TOF-PET detector readout.

The transmission line configuration of the micro-balun transformer, in a balanced-to-unbalanced signal coupling, sums the voltage response of the anode and cathode terminals of the SiPM, and the MMIC amplifier cascade provides approximately 40 dB of gain at suggested power operation (30 mW per amplifier). The circuit also incorporates an Analog Devices AD8000 high speed operational amplifier (opamp) which buffers anode voltage to be used for energy qualification in CTR measurements, similar to the method outlined in Gundacker et al (2019). A photo of the circuit assembled onto a printed circuit board (PCB) is shown in figure 1(b).

Figure 1(c) shows examples of single photon pulses generated with the SiPM and readout configuration, exhibiting high single photon SNR across a wide range of applied overvoltages to the SiPM and fast rising edges that are ⩽500 ps. In the following sections, specified power consumption only relates to the fast timing chain (MMIC cascade). The AD8000 opamp is good for benchtop testing, but its high performance is not required for energy readout of scintillation detectors. In a multichannel design, lower power components (~ 1 mW each) would be implemented, or a readout topology that does not have a voltage buffer in the configuration illustrated in figure 1(a) would be employed. Power consumption of the fast timing chain is the key metric to report, which is provided in sections 3-5. In studies where performance is reported as a function of power consumption, the voltage applied on a single line for the devices’ voltage supply, current adjustment (connected through a 3 kΩ resistor), and collector terminals was parametrically lowered. Base voltage was set by the device through internal biasing infrastructure. Thus, total power consumption for the devices was reduced by simply reducing voltage supply on a single line to the amplifiers.

### 2.2. SPTR measurements

**Figure 2.**
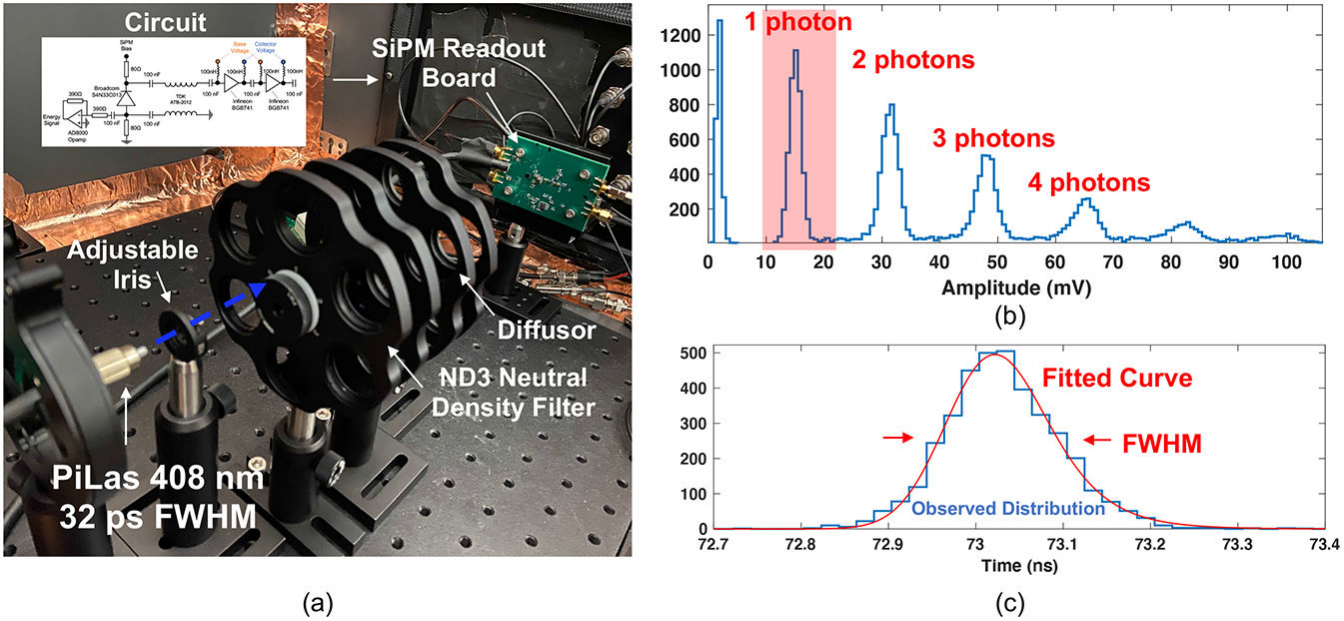
The experimental setup used to measure SPTR with the test circuit and Broadcom AFBR-S4N33C013 SiPM is shown in (a). Example pulse amplitude distribution from laser irradiation of the SiPM with the experimental setup and resulting time delay histogram (laser trigger versus leading edge threshold on photon pulses) are shown in (b) and (c), respectively.

The experimental setup used to measure achievable SPTR with the SiPM and low power electronic readout circuit is shown in figure 2(a). SPTR was quantified with a PiLas 408 nm laser with 32 ps FWHM pulse width (measured value reported from manufacturer) illuminating the test board after attenuation down to single photon light intensities and evenly spread over the sensor with an optical diffuser. Waveforms from MMICs on the test board were digitized with a Lecroy Waverunner806 digital oscilloscope at 40 GigaSamples s^−1^ (GSa s^−1^). SPTR was assessed by windowing on single photon detections (figure 2(b)), and the time delay distribution between the laser’s reference trigger pulse and the SiPM signal was fit with the convolution of a Gaussian and exponential curves (figure 2(b)), as described in Nemallapudi et al (2016). The FWHM of the resulting fit provides measured SPTR, and error was reported from the 95% confidence interval on the fitted value. SPTR was measured as a function of applied overvoltage to the SiPM and then as a function of applied voltage to the MMIC chain. Thus, the best achievable SPTR with the readout circuit was first measured, and then the tradeoff in achievable SPTR as a function of power consumption in the MMIC chain was measured to investigate the tradeoff in power and performance. Reported values for power consumption were taken from the total measured current output of a benchtop supply powering the MMIC chain, multiplied by the applied voltage. Voltage steps applied to amplifier chain were 1.25, 1.5, 1.75, 2, 2.5, and 3 V, corresponding to 5, 10, 15, 20, 30, and 60 mW of total power consumption (or equivalently 2.5, 5, 7.5, 10, 15, and 30 mW per amplifier).

### 2.3. CTR measurements

**Figure 3.**
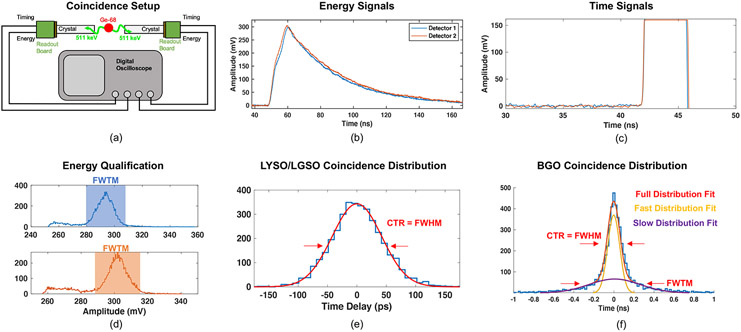
Back-to-back coincidence setup for recording CTR data is shown in (a). Example 511 keV photon pulses from the energy and timing legs of the test circuit are shown in (b) and (c), respectively, along with energy windows for event qualification in (d). Example of curve fitting for to LYSO:Ce and fast-LGSO CTR distributions is shown in (e), and example multicomponent fit for BGO distributions is shown in (f).

To demonstrate the performance of the electronic readout outlined in figure 1(a) for readout of TOF-PET detectors that employ Cherenkov photons and those that use scintillation light to derive 511 keV photon time of interaction, measurements were performed with bismuth germinate (BGO) (Shanghai Project Crystal Co., Ltd.) and also LYSO:Ce (Shanghai Project Crystal Co., Ltd.) scintillation crystal elements with 3 × 3 × 3 mm^3^ and 3 × 3 × 20 mm^3^ geometries. Measurements were also made with 3 × 3 × 20 mm^3^ fast-LGSO(0.9 mol%) crystals (Loignon-Houle et al 2017) (OXIDE Corp.). CTR was studied as a function of power consumption of the front-end signal processing chain, at an optimum applied SiPM voltage of 38 V. The specified breakdown voltage of these sensors at room temperature is 26.9 ± 0.18 V. Thus, this applied SiPM voltage corresponds to an overvoltage of 11 V. Two test boards with scintillator pixels optically coupled to the 3 × 3 mm^2^ Broadcom SiPMs were placed in a back-to-back coincidence measurement with a ^68^Ge source between them, as depicted in figure 3(a). Temperature controlled air was blown over the SiPM boards to maintain an experimental setup temperature of 20 °C. Timing and energy signals from the test boards were digitized at 40 GSa s^−1^ with the Waverunner806 digital oscilloscope and processed in offline analysis. Energy signals (figure 3(b)) from the detectors were processed with digital coincidence logic in the oscilloscope acquisition software, where event energy was determined from pulse amplitude. Validated coincidence events were selected in offline analysis from a region comprising the full-width-at-tenth-maximum (FWTM) of the 511 keV photoelectric absorption peak, as depicted in figure 3(d). Simple leading edge time pickoff was performed on digitized timing waveforms (figure 3(c)) to estimate 511 keV photon time of interaction in a sweep from 0.5 to 80 mV. Time of interaction was determined by interpolating between data points on the rising edge of the digitized waveform at the corresponding voltage threshold, mimicking an analog comparator operating on a continuous analog signal as a leading edge discriminator. No digital baseline corrections were used in leading edge time discrimination. Coincidence time distributions were built by calculating the delay between the two detector timing signals. LYSO:Ce coincidence distributions were fitted with a Gaussian function, shown in figure 3(e), and the FWHM of the fitted distribution were extracted. Distributions produced from CTR measurements with the BGO crystals were fit with a two component Gaussian function that accounts for the non-Gaussian nature of the observed coincidence distributions by incorporating separate fits for events with little or no Cherenkov photons detected by (i.e. time of interaction estimation mostly derived from BGOs luminescence yield, the ‘slow distribution fit’) and those where time pickoff is derived from the detection of Cherenkov light, as depicted in figure 3(f) (‘fast distribution fit’) (Kratochwil et al 2020). Error associated with measured CTR values for all crystals was reported from the 95% confidence intervals of the fitted parameters.

### 2.4. CTR data corrections

A rise time-based, event classification correction was also performed for the CTR measurements with BGO crystals, using the methods previously outlined in Kratochwil et al (2020). For this data correction, the time difference between leading edge time estimators for 10 mV and 80 mV crossing times were calculated for each detector and histogrammed. The rise time data was used to separate events for each detector into five regions with equal statistics. Thus, given the combination of each rise time region for both detectors, coincidence events were separated into 25 different classes, ranging from those with the fastest rise time for both detectors, to the slowest of both detectors. Classifying coincidence events in this way allows for time delay skew in leading edge time of interaction estimation to be corrected for variation in the number of detected Cherenkov photons and also that caused by time pickoff from BGO’s slower luminescence yield.

## 3. Results

### 3.1. Single photon time resolution

**Figure 4.**
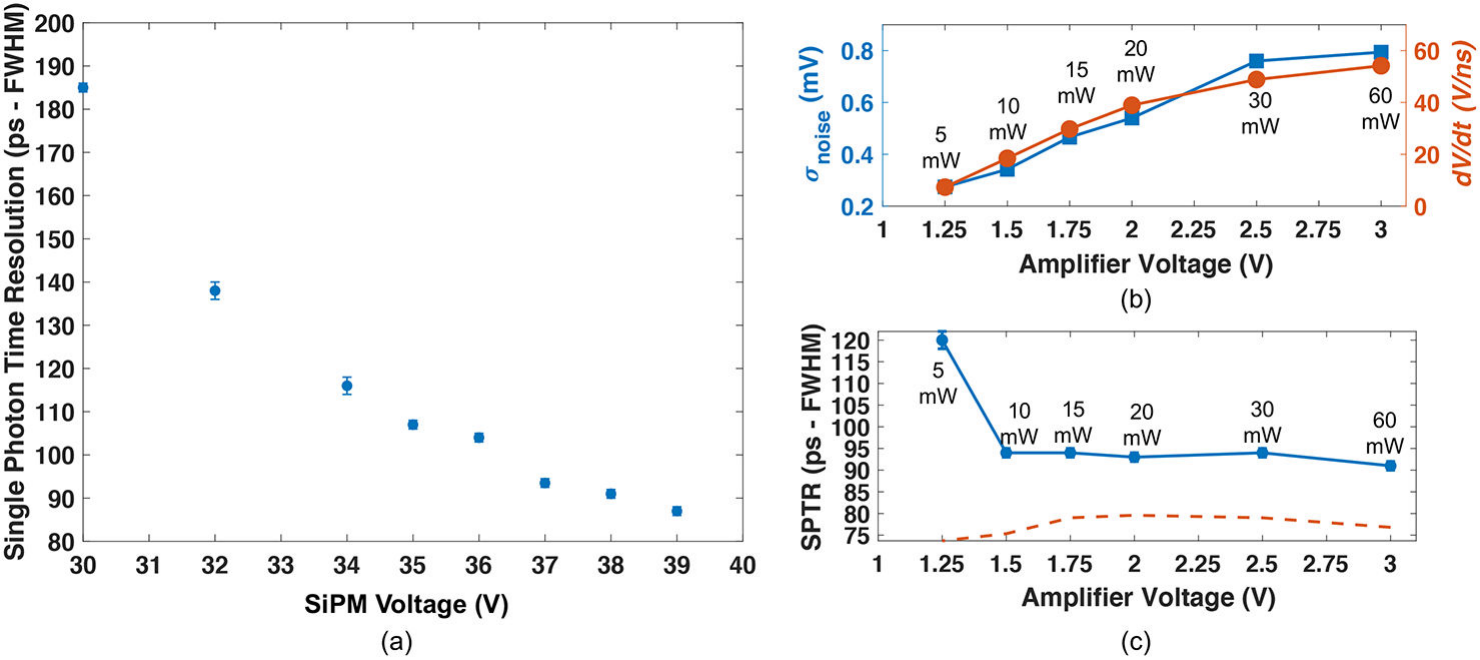
Measured SPTR with the test board is shown as a function of applied SiPM voltage in (a). In (b), electronic noise and rising edge slew are reported as a function of applied amplifier voltage to the readout circuit, and measured SPTR as a function of applied amplifier voltage is reported in (c). Total power draw from the MMIC chain at each applied amplifier voltage is also shown in (b) and (c). Calculated SPTR with the electronic noise and laser contributions to jitter removed is plotted in (c) with a dashed, orange line.

Figure 4(a) shows measured SPTR with a 3 × 3 mm^2^ AFBR-S4N33C013 SiPM on a test board with the low power readout circuit, as a function of voltage applied to the SiPM, where sub-100 ps SPTR was observed for bias voltages >36 V. These data were acquired with an 3 V applied to the MMICs, for a total power consumption of 60 mW (30 mW per amplifier). Figure 4(b) shows that reducing voltage applied to the amplifier chain proportionally reduces both electronic noise produced in the chain and also rising edge slew (SiPM bias was maintained at 38 V and slew was evaluated at half the single photon pulse amplitude, for each operating point). Thus, the timing jitter associated with the influence of electronic noise on SPTR (the ratio of electronic noise and rising edge slew) (Acerbi et al 2014a) is also minimized at reduced power consumption. Figure 4(c) shows measured SPTR at 38 V SiPM bias, as a function of power drawn from the readout circuit by lowering the voltage applied to the amplifiers and repeating the SPTR measurement. The calculated jitter from the influence of electronic noise was 37 ± 4 ps FWHM across the applied amplifier voltage range of 1.5–3 V (corresponding to 10–60 mW of total power consumption), increasing to approximately 88 ps at the lowest value of 1.25 V (corresponding to 5 mW of total power consumption). The dashed line in figure 4(c) represents the calculated intrinsic SPTR of the 3 × 3 mm^2^ Broadcom SiPM after quadratically subtracting the contribution of electronic noise and the pulsed laser source. The estimated intrinsic SPTR for the SiPM across this range of applied amplifier voltage to the readout was 77 ± 2 ps, which is consistent with the value reported in Kratochwil et al (2021) (78 ± 6) for the same SiPM using a low noise, high frequency readout. The highest applied amplifier voltage produces 60 mW of power draw, and the lowest corresponds to 5 mW of total power consumption (2.5 mW per amplifier). The implemented circuit exhibited sub-100 ps SPTR to 10 mW of power draw (1.5 V operation), where a relatively small tradeoff is observed of 120 ± 1 ps FWHM at 5 mW of power consumption (1.25 V operation).

### 3.2. Coincidence time resolution

**Figure 5.**
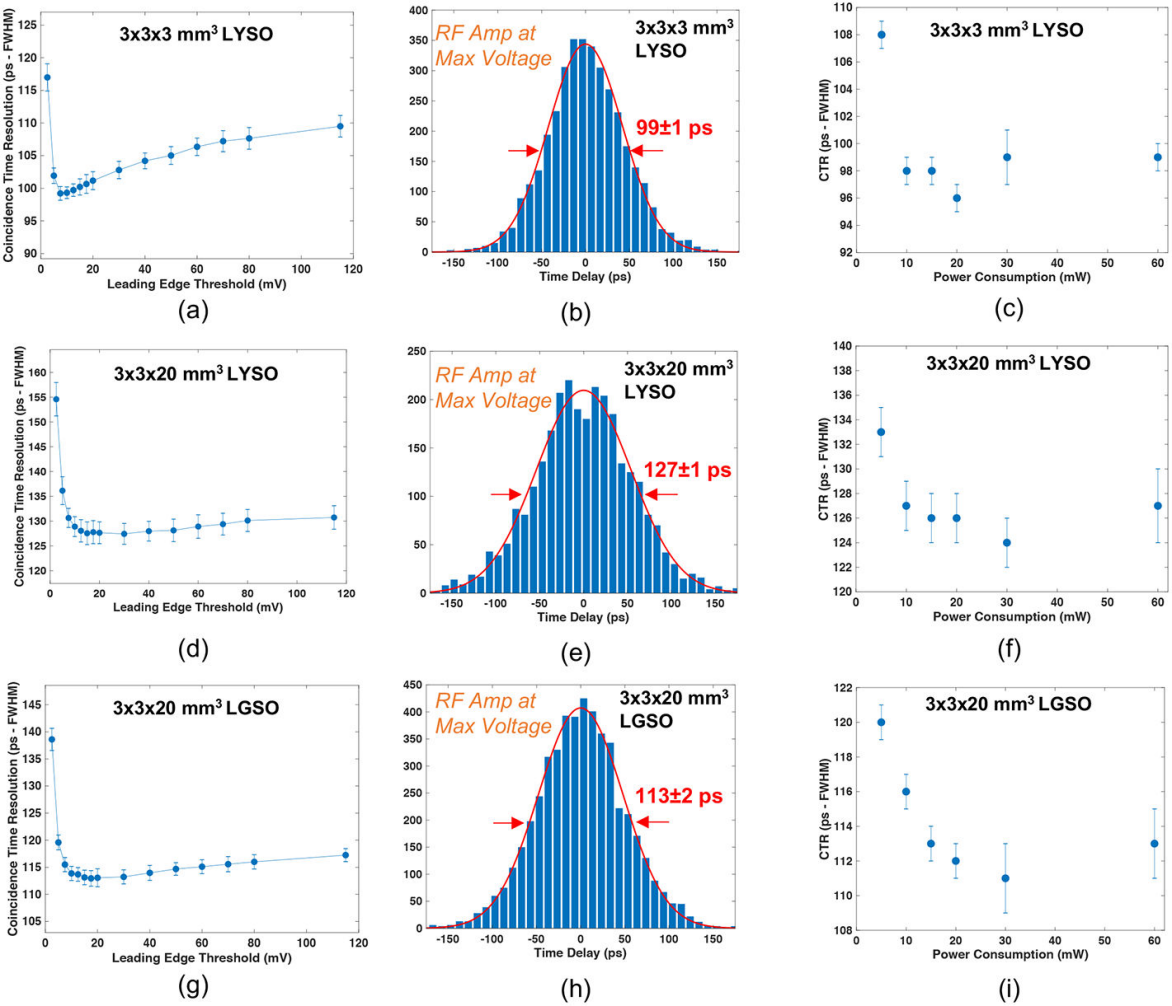
CTR at 60 mW operation is shown as a function of the leading edge time pickoff threshold for 3 × 3 × 3 mm^3^ LYSO:Ce crystals in (a), coincidence distribution at the best threshold for these detector pairs is displayed in (b), and measured CTR as a function of power consumption is shown in (c). The same plots are shown for 3 × 3 × 20 mm^3^ LYSO:Ce in (d)–(f) and 3 × 3 × 20 mm^3^ fast-LGSO in (g)–(i).

**Figure 6.**
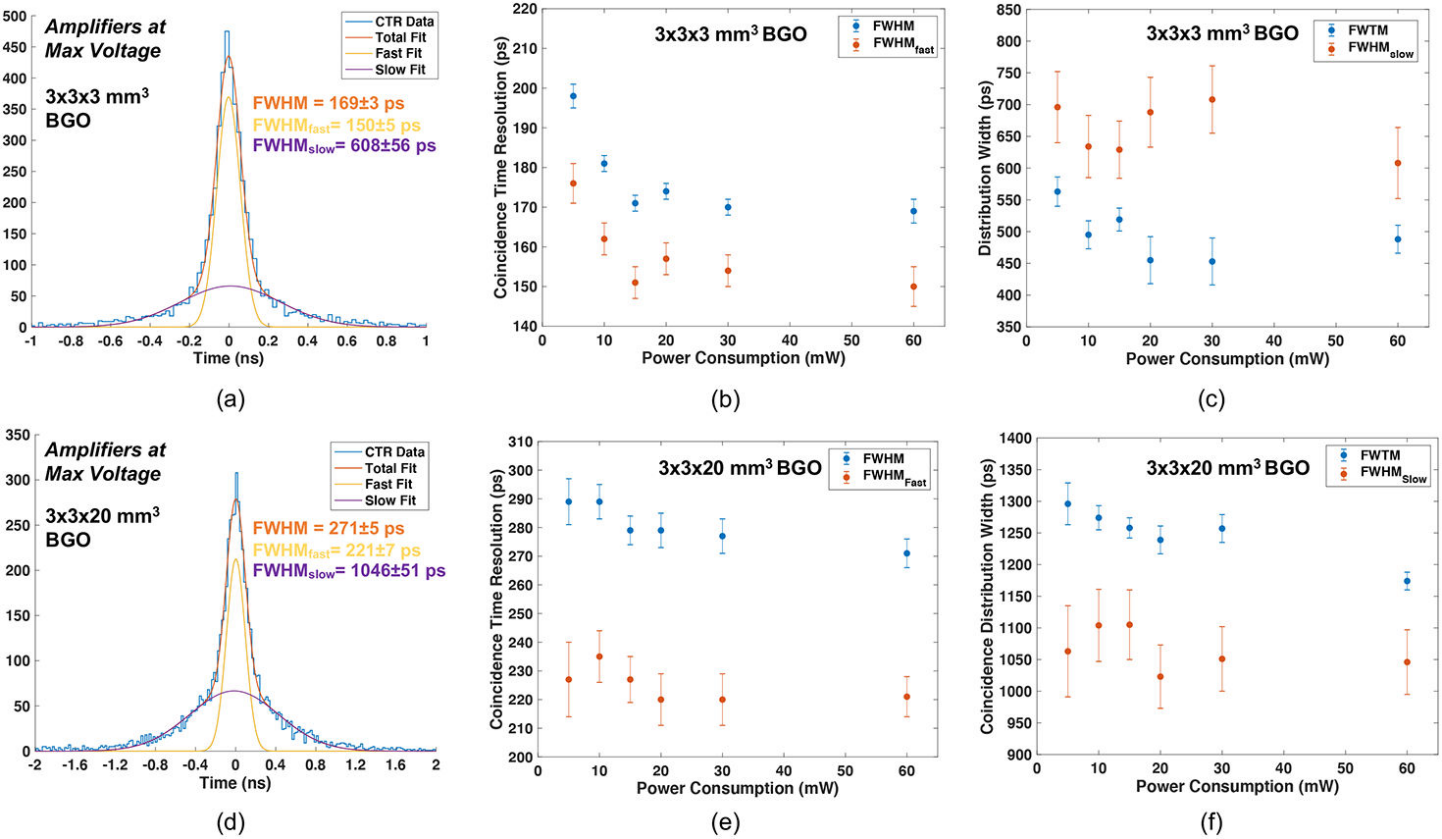
CTR distributions are shown for 3 × 3 × 3 mm^3^ and 3 × 3 × 20 mm^3^ BGO Crystals at highest amplifier voltage applied to the front-end signal processing (3 V, corresponding to 60 mW of power consumption for the timing chain) in (a) and (d). CTR and fast Gaussian FWHM are reported as a function of power consumption for 3 mm length crystals in (b) and (c). Coincidence distribution FWTM and slow Gaussian FWHM are reported as a function of power consumption for 20 mm length crystals in (e) and (f).

Figures 5(a) and (b) show measured CTR versus leading edge threshold and the CTR distribution at the optimum threshold, respectively, at the highest applied MMIC voltage (3 V, corresponding to 60 mW), for 3 × 3 × 3 mm^3^ LYSO:Ce scintillators. CTR is shown as a function of total power draw for 3 × 3 × 3 mm^3^ scintillators in figure 5(c). The same plots are shown for data from pairs of 3 × 3 × 20 mm^3^ LYSO:Ce in figures 5(d)-(f) and fast-LGSO scintillators in figures 5(g)-(i). The measured data exhibit the same performance trend as that observed for measured SPTR, where performance is maintained down to 10 mW of power draw (116 ± 1 ps for example with the 20 mm length fast-LGSO crystals), with a minor performance tradeoff at 5 mW. Thus, this readout circuit can enable PET detectors with segmented arrays that significantly advance achievable CTR, as compared to SoA systems that achieve ~200 ps FWHM. Figures 6(a) and (d) show CTR distributions for pairs of 3 × 3 × 3 mm^3^ and 3 × 3 × 20 mm^3^ BGO crystals at the highest applied amplifier voltage (3 V, corresponding to 60 mW power supply). As described in section 2.3, these distributions are fit with a multicomponent function consisting of two Gaussian curves (Kratochwil et al 2020). In figure 6, we organize the presentation of ‘fast’ and ‘slow; portions of the distribution separately. In figures 6(b) and (e), we plot the FWHM of the distribution (the CTR) and the FWHM of the ‘fast’ component of multicomponent fit, as a function of the readout circuit’s power draw for the 3 and 20 mm length crystals, respectively. Figures 6(c) and (f) plot FWTM of the coincidence distributions and the FWHM of the ‘slow’ component of fitted curve for the 3 and 20 mm length crystals. CTR ⩽ 200 ps is maintained down to 5 mW of power draw for 3 mm length BGO crystals and ⩽ 300 ps FWHM is maintained for 5 mW with the 20 mm length BGO crystals.

## 4. Discussion

Low noise, high frequency, fast circuits instrumented as front end readout for SiPM-based Cherenkov and scintillation detectors can optimize sensor time response and achievable SPTR, but implementations can also be high in power consumption and not tractable to large area prototype detectors and imaging systems. In this work, we implemented low power MMIC amplifiers into electronic readouts for SiPMs which exhibit very low noise and are capable of maintaining high gain and rising edge slew at low power operation. The combination of these capabilities produces front-end signal processing that maintains SPTR for large area SiPMs which is near the estimated intrinsic performance limits of device architecture at drastically reduced power consumption, in comparison to readout chains presented in previous works with the same performance capabilities (Cates et al 2018, Gundacker et al 2019). The readout circuit was integrated onto test boards to read out Broadcom AFBR-S4N33C013 SiPMs, which exhibited sub-100 ps FWHM SPTR down to 10 mW of power consumption and still maintained good performance, 120 ± 1 ps, at 5 mW of powerdraw. Thus, low power readout like that demonstrated in this work is excellent for fast timing in single photon counting applications and TOF-PET detectors which aim to derive accurate 511 keV photon time of interaction estimators from small populations of promptly produced optical signatures, such as Cherenkov photons.

As an example of detectors exploiting Cherenkov light for time pickoff, we presented coincidence measurements with 3 × 3 × 3 mm^3^ and 3 × 3 × 20 mm^3^ BGO crystals. CTR measured with these crystals was 169–198 ps FWHM for 3 mm length crystal elements and 271–298 FWHM for 20 mm length crystals, across a wide range of applied amplifier voltage values that produced a total power draw as high as 60 mW (30 mW per amplifier) and as low as 5 mW (2.5 mW per amplifier). These results are consistent with other CTR measurements employing the same crystal element and size, SiPM, classification-based time correction, and a different high frequency readout chain with ⩾300 mW power consumption per amplifier (Kratochwil et al 2021). In that work, 181 ± 4 ps and 261 ± 8 ps FWHM were measured for 3 and 20 mm length crystals, respectively. Thus, the implementation of low power, low noise components shown in figure 1(a) allows excellent CTR performance to be maintained at greatly reduced power consumption. BGO can be a more economical scintillation material, in comparison with lutetium-based scintillators, and it also has higher stopping power for 511 keV photons. This material has received renewed consideration for PET (Kwon et al 2016a, Brunner and Schaart 2017, Cates and Levin 2019, Kratochwil et al 2020, Gundacker et al 2020, Kratochwil et al 2021, Gonzalez-Montoro et al 2022) with advancements in photosensor technologies sensitive to near-UV light and techniques for optimizing time pickoff from low levels of Cherenkov photons, like those also employed in this work. Thus, the combination of BGO-based TOF-PET detector modules with aversion of the readout chain shown in figure 1(a) can be a pathway for high sensitivity PET systems, leveraging the increased 511 keV photon stopping power in combination with CTR equivalent to that achieved in SoA clinical systems, currently range from 200 to 400 ps FWHM (Miller et al 2015, Hsu et al 2017, van Sluis et al 2019). One ultimate realization of BGO TOF-PET detector modules could be for use in more economical PET systems with long axial extent that enable Total Body PET (TB-PET) (Cherry et al 2018).

CTR was also assessed with LYSO:Ce, a material standard to TOF-PET instrumentation research and development and also used in clinical imagers. CTR ranging from 108 to 99 ps FWHM was observed for 3 mm length crystals at 5–60 mW of power draw from the fast timing chain, and 20 mm length crystals showed 133–127 ps FWHM. As with the reported BGO values, these CTRs are consistent with values reported from measurements with high power, high frequency readout chains using the same crystal element size and SiPM. CTR for 20 mm length LYSO:Ce crystals was reported as 132 ± 2 ps inNadig et al (2022). In comparison, the test circuit shown in figure 1(a) matched this performance at 5 mW of total power consumption. Thus, readout chains like the one demonstrated in this work could significantly advance CTR of TOF-PET systems. We also measured CTR with 3 × 3 × 20 mm^3^ fast-LGSO scintillators, which showed 113 ± 1 ps FWHM CTR at 60 mW power draw and maintained sub-120 ps FWHM at 10 mW. New PET detectors employing faster scintillation materials than standard LYSO with low power, high performance readout can be a pathway towards development of large area detector modules that demonstrate ⩽ 100 ps TOF-PET with a standard PET detector design (segmented crystal arrays optically coupled 1:1 with SiPM arrays on their short end).

Overall, excellent results were achieved with all of the evaluated scintillation materials at a low power draw of 5 mW. For comparison, one of the most advanced commercial application specific integrated circuits (ASICs) for TOF-PET operates at a reported ~8 mW/channel (PETsys 20222022). The readout chain presented here is not directly comparable, as it is missing additional components, such as a comparator for time pickoff and digital back-end that includes time-to-digital converter, but it is promising that a portion of the electronics chain which typically contributes significantly to the overall required power supply is comparable to that of a high performance ASIC. Moreover, this is achieved with small, discrete element, commercial components that could feasibly be integrated into prototype PET detector modules with edge-mounted readout design, similar to implementations for other commercial PET detectors (Stolin et al 2019), or in more compact detector modules with mixed analog-digital multiplexing strategies. It is also worth mentioning that the exact implementation shown in figure 1(a) would not translate to PET detector modules and systems to be employed in simultaneous PET and magnetic resonance imaging (MRI), due to the balun’s ferromagnetic core. For detector readout designs to be operated in those environments, implementations without the balun transformer should be employed. Another interesting solution for this application might be active capacitance compensation methods (Kim et al 2020), as long as the noise, unity-gain bandwidth, and slew for the bootstrapping amplifier are not limiting in achievable electronic noise jitter.

## 5. Conclusions

We have designed and demonstrated a low power version of low noise and high frequency SiPM readout. Coupling this circuit to a Broadcom AFBR-S4N33C013 SiPM showed sub-100 ps FWHM SPTR at 10 mW of power consumption, and the readout can be biased lower with relatively minor tradeoffs in achievable SPTR (120 ± 1 ps). CTR was evaluated for 3 × 3 × 3 mm^3^ and 3 × 3 × 20 mm^3^ pixels of LYSO:Ce. The presented readout demonstrated approximately 100 ps and 130 ps for 3 mm length and 20 mm length LYSO:Ce, respectively, across a wide power consumption range of 5–60 mW. Another fast lutetium-based scintillation material, LGSO:Ce(0.9 mol%), with crystals 3 × 3 × 20 mm^3^ in size, showed CTR of 113 ± 1 ps FWHM at optimal power operation, 116 ± 2 ps at 10 mW, and still maintains excellent CTR of 120 ± 1 ps at 5 mW. BGO crystals were also evaluated with the presented readout. BGO crystal elements 3 × 3 × 3 mm^3^ and 3 × 3 × 20 mm^3^ in size achieved sub-200 and sub-300 ps FWHM CTR, respectively with 5 mW of power consumption from the fast timing chain. Readout components comprising the fast timing chain selected for this work also have very small device sizes and can feasibly be integrated into front-end readout of prototype TOF-PET detector modules. This, in combination with the high level of performance that can be achieved at low power operation, makes circuits like the one presented in this work promising for front-end readout employed in large area TOF-PET detector modules to advance SoA PET system CTR.

## Figures and Tables

**Table 1.** Compact micro-balun transformers with appropriate frequency range and port impedance for SiPM readout.

Manufacturer	Model number	Frequency (MHz)	Impedance (Ω)	Turn ratio	Size (mm^2^)
Murata	DXW21HN5011	100–1000	50	1:1	2.4
Anaren Xinger	B0322J5050AHF	300–2200	50	1:1	2.6
TDK	ATB2012	40–860/50–1200	50/75	1:1	2.4

**Table 2.** Examples of commercial, low power MMIC amplifiers with frequency response and gain appropriate for fast timing readout with SiPMs.

Amplifier^a^ manufacturer	Model number	Frequency^b^ (MHz)	Gain^c^ (dB)	Noise^c^ (dB)	Voltage (V)	Current^c^ (mA)	Size (mm^2^)
Infineon	BGA729N6	70–1000	16.3	1.05	2.8	6.3	0.77
Infineon	BGB741L7ESD	50–3500	21–16.5	1.05–1.25	3	10	2.04
Infineon	BFP840FESD	100–6000	28–24	0.55–0.75	1.8	10	1.68
NXP	BGU6101	100–3500	26.5–14.5	0.8–1.8	3	1–10	1.2
NXP	BGU7003W	40–6000	22.5–11.4	0.6–1.5	2.2–2.85	3–15	1.58

a Values for each device are presented across full range specified by manufacturer.

b Operating frequency range specified by manufacturer.

c Manufacturer measured value at the operating voltage specified for each device in the table.
